# Problems in Administrative Medicine: The Training of Institutional Porters and Male Attendants

**Published:** 1913-11-08

**Authors:** 


### PROBLEMS IN ADMINISTRATIVE MEDICINE.

### The Training of Institutional Porters and Male Attendants.

An incident which has recently occupied the attention of the management at King Edward VII.'s Hospital, Cardiff, as briefly reported already in THE HOSPITAL (October 25, 1913, p. 84), raises an old issue which every now and then crops up in institutional affairs. In hospitals, infirmaries, asylums, and all similar places for the reception of the sick, it is necessary to employ a staff of male porters and attendants to perform various duties for which female nurses are not fitted. Without any disparagement of this body of men, it is to be admitted that they come from a less educated class than do the female nurses; and it is undeniable that their training in institutions is far less complete and exacting. One reason for this is, no doubt, the fact that a large part of their duties require physical strength rather than intellectual equipment; and, indeed, herein resides the principal reason of their employment. Nevertheless, some part of their work requires a greater or less degree of technical skill and training, varying very considerably according to the exact functions assigned to the particular man. Thus many hospitals employ a male casualty-room porter or porters, whose work in some cases includes the care and preparation of dressings, sutures, instruments, anæsthetic apparatus, and in fact anything which the resident medical officers are likely to want in their daily work. Sometimes the key of the medicine and poison cupboard is entrusted to the same hands; and it is evident that such men must necessarily have acquired a considerable acquaintance with surgical principles before they could be eligible to discharge such important duties. Speaking generally, any man who reaches such a responsible post must have given beforehand to his superiors ample proof of unusual adaptability and intelligence; it is uncommon for any definite attempt to be made to train up male workers for such positions, and those who attain them generally do so by a gradual process of self-improvement gained in the school of experience and exhibited to the satisfaction of the medical officers or of the sister in charge of the department.

This, however, is a somewhat exceptional case; and the routine work of a hospital porter, so far as the patients are concerned, is confined, broadly speaking, to the removal of the sick or injured, whether from the entrance gates to the casualty rooms, from the latter to the wards, from wards to operating theatres, and so forth. Such tasks involve a great deal of handling of patients who are either unconscious or seriously ill or severely injured; and this, as doctors and nurses know well, is a business which requires not only much practice, but also a foundation of correct training and careful instruction. From time to time events happen which seem to prove a disregard on the part of hospital porters and attendants of the general principles of the transport of the sick or injured; and public impatience is then very properly manifested, often through the columns of the local press. Such an incident took place a year or two ago at one of the large London hospitals, and the latest case, as we have noted, occurred at Cardiff recently.

To find a remedy for this is not altogether a simple matter. Excellent as the character for humanity and carefulness of male workers in institutions certainly is, there are amongst them, as amongst all other sections of the community, feather-brained and even grossly careless persons; and a very small percentage of such men suffices to bring discredit upon the vast majority of those whose conduct is in every way admirable. This type no instruction will transform into thoroughly reliable servants. But we believe that more of the occasional mistakes in invalid transport made in institutions are due to lack of training than to neglect to put such training into practice. We hold that regular teaching in first aid is necessary for all the male employees of a hospital whose work does, or may, bring them into direct contact with patients. The correct management of their transport is the most important part of such a course, but by no means the whole of it; and throughout any first-aid class of this sort the fullest exposition of practical methods is essential, whereas theoretical matters may be relegated to the background.

There are several reasons why this ideal is seldom reached in our hospitals and infirmaries; but they are not really valid, and cannot be accepted as sufficient excuse. A good many hospitals offer such meagre rates of pay that strong, active, intelligent men are not content to remain for very long at a time. Hospitals could be mentioned where changes are extraordinarily frequent, simply for this reason. The shortsightedness of this policy needs no emphasis here. At many more institutions sheer conservatism is responsible. What was good enough for the last generation is not good enough, and in this case rightly, for the present one; and reform is far better before than after the occurrence of some unfortunate incident which may reflect little credit on the hospital concerned. It is not fitting that hospital porters should be allowed to pick up by gradual experience or by casual hints from their fellow-workers the proper ways to carry patients in varying circumstances, as for instance upstairs, downstairs, unconscious, and with fractures of the leg, thigh, spine, or head. Similarly, male nurses in asylums and hospitals require special training in their arduous and responsible duties. That they do not always receive it may be gathered from the evidence at a prosecution, reported in the *Sussex Daily News* some two or three weeks ago. An ex-railway guard entered the service of an asylum in June, and was immediately put on full work without an atom of training. A charge of cruelty was brought against him in September, but on the evidence, we are glad to say, the magistrates dismissed the case. The point we would press is that such things should be impossible, and that much greater care should be taken over the training of male workers in institutions.

